# Coding definitions of participant religious, non-religious and spiritual beliefs in the Avon Longitudinal Study of Parents & Children (ALSPAC).

**DOI:** 10.12688/wellcomeopenres.20209.1

**Published:** 2023-11-16

**Authors:** Yasmin Iles-Caven, Steven Gregory, Sarah Matthews

**Affiliations:** 1 Bristol Medical School (Population Health Sciences), University of Bristol, Bristol, England, UK

**Keywords:** ALSPAC, religion, non-religious, spirituality, denominations, alternative beliefs, intergenerational, time trends

## Abstract

Mainstream religious beliefs and behaviours have been shown to have positive effects on health and well-being, but there has been increasing secularisation in the West over time. With concurrent increases in those stating they have no religion (the ‘nones’) there are increasing numbers now describing themselves as humanist, ‘spiritual but not religious’ or who have sought alternative forms of belief. Others have formed their own beliefs using elements of different belief systems. This trend is reflected in ALSPAC data with larger proportions considering themselves as ‘nones’, agnostic or atheist, and about 3% of parent participants consistently stating they had ‘other’ beliefs. The main aim of this paper is to describe the coding of the Christian denominations, world religions, non-mainstream beliefs (NMB) and non-religious groups derived from the text-based data collected from the original mother and partner cohorts (G0). This spans a period of ~28 years from pregnancy onwards. We also describe the coding of text-based responses from their offspring (G1) collected at ages 27+ and 29+. The creation of this coded data will enable researchers to compare between the Christian denominations and/or other belief groups taken from two generations alongside the rich resource of physical and mental health, behavioural and social data that exists within ALSPAC.

## Background

With each successive generation, there is evidence that, particularly in the West, people are becoming more and more secular ( Chaves, 2017; ONS, 2012; Pew Forum on Religion and Public Life, 2017; Twenge *et al.*, 2015). There is ample literature indicating that organised religious belief and behaviours have been shown to have positive associations with outcomes such as coping with serious illness, traumatic life events and improved mental well-being ( *e.g.,* Idler & Kasl, 1997; McFadden, 1995; Ness & Wintrob, 1980; Pargament, 1990; Seligman, 1991). Concurrently, there has been growing interest in what the non-religious believe and any beneficial or detrimental health effects.

With the move away from mainstream religion, many people now state they are agnostic, atheist (see Iles-Caven *et al.*, 2019), non-religious or ‘spiritual but not religious’ (SBNR) and many seek alternatives. As Wixwat and Saucier concluded in their review ( 2021) “Making sense of the ‘spiritual but not religious’ identification is a somewhat complex endeavor; there are multiple motives involved, and multiple meanings for spirituality. Preference for this identification appears to reflect some personality and especially cultural and demographic factors. At least in Western populations, spiritual tendencies (including those of a mystical nature) can be differentiated from conventionally religious tendencies, but spirituality is itself heterogeneous.” In addition, there are those who have sought alternatives in non-mainstream beliefs (NMB **s**); New Religious Movements (NRMs) such as Scientology and the Unification Church; or other forms of belief including blending elements from various religions or philosophical treatises; humanist approaches, or those considered New Age. In this paper, we define NMBs as those world religions uncommon in the UK at the time of enrolment in ALSPAC (Avon Longitudinal Study of Parents and Children) as well as New Age, Pagan, NRMs, the spiritual but not religious, and those with their own beliefs.

Comparison of parent (G0) ALSPAC data with UK Census data ( ONS, 2012) for the Avon area and for England and Wales are shown in Table 1. This shows that the decline in Christianity over the last two decades shown in local and national figures, is reflected in ALSPAC along with a concurrent increase in those stating no religion (the ‘nones’). Mainstream world religions are under-represented in both ALSPAC and Avon compared with England and Wales. Interestingly, there is a steady increase in NMBs across all areas, but ALSPAC participants had the highest levels ( *e.g*., for 2021, 3.8% *vs*. 0.6% nationally). Note that ALSPAC participants were more likely to state their beliefs than UK Census participants. Within ALSPAC we found higher numbers reporting an eclectic mix of beliefs and practices including New Age, Pagan, and many who profess ‘their own beliefs’, but fewer affiliated with mainstream world religions common in the UK (Islam, Hindu, Sikh, Judaism, Buddhism). This, too, is reflected in census data for Bristol in 2011 and 2021 ( Table 2). To assist the reader, we have created a glossary with brief descriptions of all beliefs found within ALSPAC and the Bristol area (Extended data: Appendix 2), each followed by the new umbrella code under which they fall in the coding framework (Extended data: Appendix 1).

**Table 1.** Comparison of beliefs (%) in ALSPAC parents (G0) with Avon, National Census and US data. Note that the religion question is optional in the census and was first asked in 2001. Avon = Bristol City, South Gloucestershire and North Somerset figures combined.

Stated beliefs	ALSP 1990-1	ALSP 2000	ALSP 2020	Avon 2001	Avon 2011	Avon 2021	Engl & Wales 2001	Engl & Wales 2011	Engl & Wales 2021	Pew USA 2014
**N**	**22,471**	**11,648**	**6,965**	**814,820**	**893,567**	**979,623**	**52.0m**	**56.0m**	**59.6m**	**35,000**
Not stated	2.8	4.2	1.2	8.3	8.1	6.5	7.7	7.2	6.0	-
None	20.0	17.9	30.9	20.0	33.8	48.4	14.8	25.1	37.2	23.4 #
Christian	73.2	74.4	62.8	68.0	53.8	38.9	71.8	59.3	46.2	70.8
Other *	1.3	0.9	1.3	1.9	4.1	5.6	5.4	8.0	10.1	4.2
NMBs	3.4	2.4	3.8	0.3	0.5	0.7	0.3	0.4	0.6	0.7 #

*Combined Buddhism, Muslim, Hindu, Sikh, Judaism. #For the US data only, None also includes 0.6% who ‘don’t know’; the NMB figure combines “other world religions, Unitarian and other liberal faiths, Native American, and New Age/Pagan”.

**Table 2.** Variety of beliefs from the city of Bristol Census data.

Stated beliefs	2011 Bristol N = 428,234	2021 Bristol N = 472,400	Stated beliefs	2011 Bristol	2021 Bristol
Religion not stated	34,782 (8.1)	30,706 (6.5)	Jain	30	30
No religion	154,096 (36.0)	241,800 (51.2)	Heathen (pagan, neo-pagan)	21	34
Agnostic	506 (0.1)	610 (0.1)	Zoroastrian	21	30
Atheist	476 (0.1)	207 (0.0)	Shamanism	17	11
Christian	200,254 (46.8)	152,126 (32.2)	Occult	15	15
Islam	22,016 (5.1)	31,776 (6.7)	New Age	13	1
Hindu	2,712 (0.6)	3,545 (0.7)	Traditional African Religion	13	9
Buddhist	2,549 (0.6)	2,710 (0.6)	Scientology	12	9
Sikh	2,133 (0.5)	2,247 (0.5)	Shintoism	11	16
Judaism	777 (0.2)	1,228 (0.3)	Theism	11	7
**Total NMBs:**	**5,363 (1.2)**	**3,662 (0.8)**	Deist	10	15
Jedi Knight	2,310	0	Universalist	9	6
Pagan	575	820	Witchcraft	7	13
Mixed religion	415	133	Animism	5	19
Spiritualist	376	252	Free Thinker	5	0
Spiritual	269	587	Reconstructionist	5	10
Rastafarian	243	148	Church of All Religions	4	1
Humanist	190	121	Ravidassia	4	0
Other religions	189	787	Realist	4	3
Wicca	99	99	Thelemite	4	5
Baha’i Faith	83	92	Eckankar	3	3
Taoist/Daoist	81	71	Native American Church	3	1
Believe in God	80	28	Vodun	2	0
Heavy Metal	61	0	Brahma Kumari	1	1
Druid	47	22	Confucianist	1	0
Pantheism	44	54	Mysticism	1	3
Own belief system	34	38	Unification Church (Moonies)	1	1
Satanism	34	114	Yazidi *	0	42
Alevi *	0	11			

*Relatively recent refugee populations

A significant amount of research into religious beliefs has emanated from the USA, considered a much more religious country than the UK, but comparisons could be made with ALSPAC data. According to the Pew Forum on Religion and Public Life (2017) (cross-sectional) sweep of 2014, the majority of the population (70.8%) were Christian (of which 20.8% were Catholic, 0.5% Orthodox, 1.6% Mormon, 0.8% Jehovah’s Witness, 25.4% Evangelical Protestant and 14.7% Mainline Protestant). Other faiths comprised: 1.9% Judaism, 0.9% Muslim, 0.7% Buddhism, 0.7% Hinduism and 0.3% other World Religions. New Age comprised 0.4% of the population, of which 0.3% were Pagan or Wiccan. The proportion of world religions are on a par with Avon (in 2011, the closest timepoint); NMB beliefs (0.7%) are broadly similar to the rates found in Avon and in England and Wales, but the US data also included ‘Unitarians and other liberal faiths’ in this category (1%). 22.8% were ‘nones’ compared with the UK (25.1%) in 2011 (see Table 1).

The aim of this paper is to describe the coding of text relating to beliefs from parents enrolled in the Avon Longitudinal Study of Parents and Children (ALSPAC), collected initially antenatally in 1991/92, at five, nine and 28 years after the birth in 2020, and from the offspring at ages 27 and 29. At the 2020 sweep the parents (G0) and their offspring (G1) were also asked two additional belief questions which also had the option for a text response (see Methods below). Current beliefs were also asked again of both cohorts just prior to the pandemic (2021) and post-pandemic (2022). 

The text responses revealed a large number of Christian denominations; as well as non-religious or non-theist beliefs as van Mulukom *et al.* (2023) have also reported in their study carried out across ten countries.

## Methods

### Participants

***The Avon Longitudinal Study of Parents and Children (ALSPAC)***

All pregnant women (G0 cohort) resident in the Avon area of south-west England with expected dates of delivery between April 1991 and December 1992 inclusive were invited to enrol in ALSPAC. The recruitment area comprised three of the District Health Authorities within the county of Avon (Bristol & Weston, Frenchay, and Southmead). Avon was disbanded in 1996 and the areas split between the city of Bristol, North Somerset, South Gloucestershire and an area of Bath and Northeast Somerset (see Boyd *et al.*, 2013 for a map; and Avon (county) - Wikipedia for more details about Avon). The initial number of pregnancies enrolled was 14,541, resulting in 13,988 infants who were alive at age 12 months. Additional phases of enrolment are described in more detail in the cohort profile papers ( Boyd *et al.*, 2013; Fraser *et al.*, 2013; Major-Smith *et al.*, 2022; Northstone *et al.*, 2019). The total sample size for analyses using any data collected after the age of seven is 15,447 pregnancies, resulting in 15,658 fetuses. Of these, 14,901 children (G1 cohort) were alive at 1 year of age. Some women enrolled with one or more pregnancies, consequently the number of unique mothers who were enrolled was 14,833 in total. G0 partners were invited to complete questionnaires by the mothers at the start of the study but they were not formally enrolled at that time. 12,113 G0 partners have been in contact with the study by providing data and/or formally enrolling when this commenced in 2010. 3,807 G0 partners are currently enrolled ( Northstone *et al.*, 2023). Since 2014, study data are collected and managed using REDCap electronic data capture tools hosted at the University of Bristol. REDCap (Research Electronic Data Capture) is a secure, web-based software platform designed to support data capture for research studies ( Harris *et al.*, 2009). For those participants who prefer to complete paper versions these are also offered. Completed paper questionnaires are scanned into electronic data using Teleform data capture software. Please note that the study website contains details of all the data that is available through a fully searchable data dictionary and variable search tool.

Ethical approval for the ALSPAC study was obtained from the ALSPAC Ethics and Law Committee (ALEC; IRB00003312) and the Local Research Ethics Committees. Detailed information on the ways in which confidentiality of the cohort is maintained may be found on the study website. All methods were performed in accordance with the relevant guidelines and regulations. Informed consent for the use of data collected via questionnaires and clinics was obtained from participants following the recommendations of the ALSPAC Ethics and Law Committee at the time. Study members have the right to withdraw their consent for elements of the study or from the study entirely at any time. Consent for biological samples was collected in accordance with the Human Tissue Act (2004). Details of the approvals obtained are available in full from the Ethics pages of the study website ( http://www.bristol.ac.uk/alspac/researchers/research-ethics/) ( Birmingham, 2018).

Basic demographics of the parent (G0) cohorts are presented in Table 3.

**Table 3.** Demographics of ALSPAC parent cohort during the antenatal period.

Sociodemographic *variable name*	Mothers N	%	Partners N	%
**Age at delivery** *mz028b; partner_age*				
<25	4676	30.7	1832	15.2
25-34	9153	60.2	7714	63.8
35+	1378	9.1	2539	21.0
	15207		12085	
Range (years)	<16-41		<16-65+	
**Ethnicity** *c800, c801*				
White	11902	97.4	11493	96.0
Non-white	320	2.6	476	4.0
	12222		11969	
**Highest Educ** *c645a, pb325a*				
<0 level	7969	64.7	5143	52.6
A level+	4344	35.3	4639	47.4
	12313		9782	
**Marital status** *a525 pa065*				
Currently married	9996	74.8	6932	81.8
Not currently married	3369	25.2	1541	18.2
	13365		8473	
**Social class** * c755, c765*				
Non-manual (I-IIINM)	7990	80.1	6066	55.6
Manual (IIIM-V)	1983	19.9	4787	44.1
	9973		10853	
**Sex of study child at birth** *kz021*				
Male	7689	51.1		
Female	7355	48.9		
	15044			
**Housing tenure** *a006*			-	-
Owner occupied	9732	73.1		
Rented/all other	3577	26.9		
	13309			
**Urban/Rural** *Jan1993ur01ind_m*			-	-
Urban	11676	90.2		
Town & fringe	528	4.1		
Village	531	4.1		
Hamlet/isolated dwelling	202	1.6		
	12937			
**IMD** *Jan2014md2010q5*			-	-
1 least deprived	3936	30.5		
2	2939	22.8		
3	2281	17.7		
4	2108	16.3		
5 most deprived	1631	12.6		
	12895			

### Data collection

The religious/spiritual belief and behaviour (RSBB) questions were administered during pregnancy, at five years and nine years post-delivery to the G0 parent cohorts and ~28 years post-delivery to both the G0 parent and G1 offspring cohorts in 2020; they are detailed elsewhere ( Iles-Caven *et al.*, 2019; Iles-Caven *et al.*, 2021; Iles-Caven *et al.*, 2022). Subsequently, the RSBB questions were asked again at ~29 years (G1s were administered from December 2021 and the G0s from January 2022) in order to capture changes in beliefs post-pandemic.

The tick box (self-coded) options for the question ‘What sort of faith/belief would you say you have nowadays?’ are shown in Box 1 (a) and (b).

**Box 1:** 

(a) Pre-2020 Codes	(b) 2020 onwards
0 None 1 Church of England 2 Roman Catholic 3 Jehovah’s Witness 4 Christian Science 5 Mormon *6 Other Christian (please describe)* 7 Judaism 8 Buddhism 9 Sikhism 10 Hindu 11 Muslim 12 Rastafarianism (latterly coded to 304) *13 Other (please describe)*	1 Church of England 2 Roman Catholic 3 Jehovah’s Witness 4 Methodist tradition 5 Baptists/Evangelical *6 Other Christian (please describe)* 7 Judaism 8 Buddhism 9 Sikhism 10 Hindu 11 Muslim 12 Rastafarianism 13 None *14 Other (please describe)*
(c) Other Christian - umbrella codes	(d) Other beliefs – umbrella codes
101 Baptist/Evangelical 102 Methodist 103 Presbyterian 104 Pentecostal 105 Quaker 106 Orthodox churches 107 Lutheran 108 Adventists 109 Protestant NOS 110 Non-Trinitarian denominations 111 Non-Conformists 112 Christian, NOS (includes mixed denominations)	201 Spiritualists 202 New Age 203 Eastern religions/beliefs (excluding 8-11 above)/Philosophies. 204 Western Philosophies/Humanism 205 Pagan 206 NRM (New Religious Movements) 301 Spiritual (Spiritual but not religious, SBNR) 302 Higher Power(s) 303 Miscellaneous/Own beliefs 304 Syncretism 305 Mixed households 306 Personal choice 307 Magic 401 Agnostic 402 Atheist 501 Niche 601 Unclassifiable

At the 2020 sweep two additional questions were asked of the G0s and the G1s, which offered a text response:

‘Were you brought up in this faith (including ‘none’)? Yes, this faith / No, If no, what faith were you brought up in if any?’

G0 cohort: ‘Did you bring your child(ren) up in your current faith (including none)? Yes, this faith / No, If no, what faith, if any, did you bring your child(ren) up in?’

G1 cohort: ‘Would/Are you bringing your child(ren) up in your current faith/belief (including none)? Yes, this faith / Not sure/ Not applicable / No, If no, what faith would you/are you bringing up your children in, if any?’

These text responses have been coded using the same methodology, with the addition of a few extra codes to take into account those G1s brought up in mixed households (e.g., Catholic + Hindu); and wording along the lines of ‘allowed to make up my own mind’ (Personal choice).

In 2021-22, Life at 29+ was administered to the G1 cohort (from December 2021) and a similar questionnaire administered to the G0s (from January 2022). These requested information on beliefs since the beginning of the COVID-19 pandemic in order to elicit changes in belief during it: ‘What sort of faith/belief would you say you have nowadays?’; the extent to which they considered themselves to be religious/spiritual nowadays; significant losses and gains in faith during the pandemic and so on. The types of faith/belief have been coded as for the earlier questionnaires.

### Coding methodology

The coding for the earlier sweeps (Antenatal, 5- and 9-years post-delivery) differ slightly from the 2020 questionnaire. The alteration for 2020 (and for any subsequent data collection), reflected the large numbers who had written ‘Baptist’ or ‘Methodist’ under ‘Other Christian’ and the very small numbers who had ticked ‘Christian Science’ or ‘Mormon’ (see Box 1 a and b). In order to protect confidentiality (where cell counts were <5) and make coding more manageable about 150 denominations and other beliefs were condensed into a total of 12 Christian and 17 ‘other’ umbrella codes). Box 1 c and d shows the final ‘umbrella’ codes for the Christian denominations and NMBs respectively. The final coding schedule is presented as Appendix 1 (Tables 1a-1c) and describes the contents under each umbrella code. For example, the Orthodox umbrella includes the responses: Christian Orthodox; Coptic church; Eastern, Greek, Polish, Romanian and Russian Orthodox, and Orthodox not otherwise stated; The Pagan umbrella includes ancestor worship, religions of antiquity, Shamanism, Druids, Heathens, Norse, Wicca etc.

***(a) Christianity***

**Figure 1.**
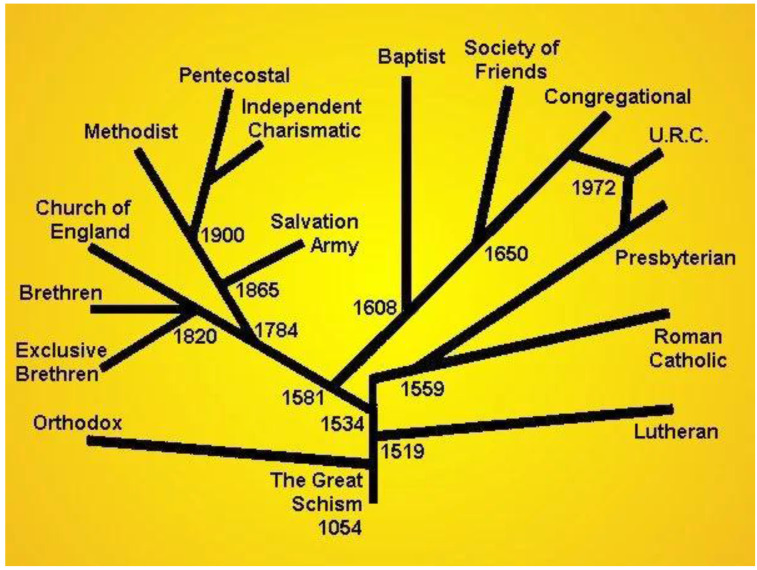
“ History of Christian denominations.” by JodiP. Attribution-ShareAlike 4.0 International (CC BY-SA 4.0).

The majority of respondents in ALSPAC are Christian (mainly Church of England (CofE)), but on examining the text responses to ‘Other Christian’ we found almost 50 denominations including various Orthodox, Lutheran, Pentecostal and Presbyterian churches. An attempt was made to condense all the responses into fewer codes, which involved investigation to ascertain the best category using a historical overview of the Christian Church (see Figure 1), and how various denominations evolved. However, there are many churches which could fit under more than one category such as ‘Charismatic Free Church’ and so we have stated which denominations/churches are included under each code. Although we have chosen to group Baptists and Evangelical Not Otherwise Stated under one code, almost any church could also be evangelical (e.g. Evangelical Pentecostal) and therefore were coded under the main denomination (Pentecostal in this case). Some respondents stipulated more than one denomination (e.g. “Baptist, Presbyterian and Church of England” or “Catholic/CofE”) and these have been coded under ‘Other Christian’ (see Extended data, Appendix 1, Table 1b).

The main reason for reducing the number of codes was to protect confidentiality where there were <5 members of that denomination, but also to facilitate research between denominations, for example: Church of England vs Pentecostal. The <5 limit was deemed by ALSPAC to be the maximum number by which it could conceivably be possible to identify a participant. Therefore, frequencies in the tables that are 0-4 are denoted <5.

***(b) World Religions***

The options for world religions reflected those most prevalent in Avon at the first enquiry in 1990/91 (Judaism, Buddhism, Sikhism, Islam, Hindu, Rastafarianism). As numbers who professed to be Rastafarian were very low, these have been included under the ‘Syncretic’ umbrella (see Appendix 1, Table 1c). Currently (2023), there are five Buddhist centres, one Hindu temple, four Sikh temples, five mosques and two synagogues within Bristol and one mosque in Weston-Super-Mare that service the Avon area. A limitation and criticism of ALSPAC has been the lack of ethnic diversity in those enrolled in the study, despite considerable attempts to recruit them with multi-lingual recruiting material ( Iles-Caven *et al.*, 2020). This is reflected in the low numbers of those affiliated with the world religions reported here.

***(c) Non-mainstream beliefs (NMBs)***

Text responses to the ‘Other’ beliefs were even more complex and varied than the Christian denominations, with over 100 variations. The final umbrella coding model is presented in Box 1 above, and detailed in Appendix 1, Table 1c. See Appendix 2 for glossary of beliefs.

As well as formal religions such as Jainism, Zoroastrian, Shinto, Baha’i Faith and Taoist we found an eclectic mix of New Age, Pagan, the Occult, beliefs of the indigenous peoples of North America and Oceania, followers of New Religious Movements (NRMs) or particular philosophers, humanists, the ‘spiritual but not religious’ and many who had their own beliefs. This latter group, by far the largest, posed some challenges with mixtures such as “Humanist/Buddhist/New Age and Spiritual” and “Buddhist/Taoist/Christian” or personal beliefs: “My own”, “Nature”, “the Universe”. Again, considerable effort was put into coding them to meaningful categories whilst protecting participant confidentiality. See Appendix 1, Table 1c for what is included under each umbrella grouping.

There were many instances where respondents had ticked ‘Other Christian’ or ‘Other’ and written atheist or agnostic in a text line. These were recoded to Atheist or Agnostic. These are additional to those ticking ‘none’.

The spiritual vs. spiritualists: These have very different meanings (see Huss, 2014 for an interesting discussion on the modern meaning of spirituality), but it is likely that some respondents may not have realised that difference. A potential problem found on coding was those stipulating they were ‘spiritual’ or ‘spiritual but not religious’ compared with those stating ‘spiritualist’/‘spiritualism’ in the ‘Other’ rather than in the ‘Other Christian’ text box. Some stipulated, ‘Spiritualist Church’ and these are coded under the Pentecostal umbrella.

Please note that all the groupings for coding were devised by the author (YIC) based on best fit and to protect the confidentiality of participants where cell counts were <5 and some readers may disagree with these decisions.

## Observations

Previous research using ALSPAC parent data, where we have 3600 mothers and 1200 partners with data at all four timepoints, have shown that the Church of England had the greatest loss over time whereas Roman Catholics were much more likely to remain affiliated to the Holy See ( Major-Smith *et al.*, 2023). We also previously reported strong sex differences (all P<0.001) in religious beliefs: 49.9% of women stated that they believed in God, or a divine power compared with 37% of men ( Iles-Caven *et al.*, 2019). For all denominations and NMBs, ALSPAC women tended to have slightly higher frequencies than their partners, with the exception of Western Philosophies/Humanists ( Tables 4a, b). This confirms previous research that women tend to be more religious/spiritual (e.g., Trzebiatowska & Bruce, 2012). For the G1 cohort, over 5% stated that they had NMB beliefs ( Table 4c) at both ages 27+ and 29+.

**Table 4a.** G0 Mothers type of faith over time in ALSPAC (as per new text coding system). Cell counts of <5 may include values of zero.

G0s Code/Belief	Mothers				
Age of Index child Dates of data collection *Variable name*	AN 1991/92 *D813*	5y PP 1996/97 *K6243*	9y PP 2000/01 *P4044*	27+yr PP 2020 *Y3040*	30+yr PP 2022 *Z5540*
*Belief in God/Divine power*					
Yes	6160 (49.9)	4141 (46.5)	3776 (48.2)	2082 (43.5)	1817 (38.9)
No	1838 (14.9)	1745 (19.6)	1369 (17.5)	1270 (26.6)	1443 (30.9)
Not sure	4353 (35.2)	3018 (33.9)	2682 (34.3)	1429 (29.9)	1416 (30.3)
Total of those answering belief questions:	12231	8761	7672	4732	4649
0 None	1818 (14.9)	1283 (14.6)	1189 (15.5)	1285 (27.2)	1761 (37.8)
1 Church of England	7837 (64.1)	5556 (63.4)	4637 (60.4)	2315 (48.9)	1929 (41.5)
2 Roman Catholic	1018 (8.3)	691 (7.9)	593 (7.7)	361 (7.6)	280 (6.0)
3 Jehovah’s Witness	55 (0.4)	42 (0.5)	38 (0.5)	20 (0.4)	17 (0.4)
4 Christian Science	15 (0.1)	17 (0.2)	11 (0.1)	N/A	N/A
5 Mormon	28 (0.2)	22 (0.3)	14 (0.2)	N/A	N/A
*Other Christian - not specified*	99 (0.8)	100 (1.1)	425 (5.5)	16 (0.3)	61 (1.3)
101/5 Baptist/Evangelical	237 (1.9)	190 (2.2)	107 (1.4)	184 (3.9)	158 (3.4)
102/4 Methodist	271 (2.2)	206 (2.4)	88 (1.1)	189 (3.9)	170 (3.7)
103 Presbyterian	82 (0.7)	54 (0.6)	45 (0.6)	21 (0.4)	13 (0.3)
104 Pentecostal	46 (0.4)	40 (0.5)	24 (0.3)	8 (0.2)	<5
105 Quaker	13 (0.1)	15 (0.2)	18 (0.2)	10 (0.2)	13 (0.3)
106 Orthodox	22 (0.2)	14 (0.2)	15 (0.2)	6 (0.1)	6 (0.1)
107 Lutheran	8 (0.1)	<5	<5	<5	<5
108 Adventist	10 (0.1)	<5	5 (0.1)	<5	<5
109 Protestant NOS	35 (0.3)	21 (0.2)	26 (0.3)	5 (0.1)	<5
110 Non-Trinitarian	17 (0.1)	10 (0.1)	8 (0.1)	11 (0.2)	8 (0.2)
111 Non-Conformist	5 (0.0)	11 (0.1)	<5	<5	<5
112 Christian NOS	124 (1.0)	127 (1.4)	125 (1.6)	27 (0.6)	32 (0.7)
7 Judaism	13 (0.1)	12 (0.1)	10 (0.1)	5 (0.1)	5 (0.1)
8 Buddhist	27 (0.2)	20 (0.2)	31 (0.4)	35 (0.7)	38 (0.8)
9 Sikh	16 (0.1)	6 (0.1)	5 (0.1)	11 (0.2)	5 (0.1)
10 Hindu	23 (0.2)	13 (0.1)	6 (0.1)	<5	-
11 Muslim	55 (0.4)	22 (0.3)	18 (0.2)	11 (0.2)	10 (0.2)
*Other – not specified*	30 (0.2)	39 (0.4)	20 (0.3)	36 (0.8)	64 (1.4)
201 Spiritualist	39 (0.3)	27 (0.3)	35 (0.5)	49 (1.0)	20 (0.4)
203 Eastern Philosophies	10 (0.1)	5 (0.1)	7 (0.1)	5 (0.1)	<5 #
204 West Philosophies/ Humanists	15 (0.1)	11 (0.1)	12 (0.2)	6 (0.1)	11 (0.2)
202 Pagan + 205 New Age *	13 (0.1)	17 (0.2)	18 (0.2)	22 (0.4)	22 (0.5)
206 NRMs	5 (0.0)	<5	<5	<5	<5
301 Spiritual (SBNR)	8 (0.1)	9 (0.1)	16 (0.2)	40 (0.8)	55 (1.2)
302 Belief in Higher Power(s)	33 (0.3)	17 (0.2)	16 (0.2)	6 (0.1)	16 (0.3)
303 Own/Misc beliefs + 307 Magic *	78 (0.6)	40 (0.5)	37 (0.5)	30 (0.6)	30 (0.6)
304 Syncretic	8 (0.1)	<5	<5	<5	<5
401 Agnostic	36 (0.3)	29 (0.3)	17 (0.2)	9 (0.2)	8 (0.2)
402 Atheist	69 (0.6)	75 (0.9)	36 (0.5)	<5	<5
501 Niche + 601 Unclassifiable *	13 (0.1)	8 (0.1)	10 (0.1)	6 (0.1)	<5

#Includes Hindu at this time point; *amalgamated due to small cell counts AN = Data collected during pregnancy; PP = post-partum, i.e. responses at age in years of index child. NRMs = New religious movements; SBNR = spiritual but not religious; NOS = not otherwise stated N.B. Agnostics and Atheists are those that have stated such in text form and are additional to Q1 on belief in God or a divine power (Yes, No, Not sure)

**Table 4b.** G0 Partners type of faith over time in ALSPAC (as per new text coding structure). Cell counts of <5 may include values of zero.

G0s Code/Beliefs	Partners				
Age of Index child Dates of data collection *Variable name*	AN 1991/92 *pb153*	5y PP 1996/97 *ph6243*	9y PP 2000/01 *pm4044*	27+yr PP 2020 *FPC3040*	30+ yr PP 2022 *FPD5540*
*Belief in God or Divine Power*	N=	N=	N=	N=	N=2057
Yes	3621 (37.0)	1505 (33.6)	1275 (35.3)	654 (30.0)	555 (27.0)
No	2801 (28.6)	1406 (31.4)	1149 (31.9)	986 (45.3)	997 (48.5)
Not sure	3376 (34.5)	1573 (35.1)	1183 (32.8)	538 (24.7)	505 (24.6)
Total of those answering belief questions	N=9641	N=4367	N=3499	N=2147	N=2045
None	2404 (24.9)	1025 (23.5)	865 (24.7)	863 (40.2)	1024 (50.1)
1 Church of England	5281 (54.8)	2466 (56.5)	1854 (53.0)	889 (41.4)	699 (34.2)
2 Roman Catholic	723 (7.5)	323 (7.4)	278 (7.9)	137 (6.4)	95 (4.6)
3 Jehovah’s Witness	31 (0.3)	22 (0.5)	13 (0.4)	8 (0.4)	10 (0.5)
4 Christian Science	13 (0.1)	7 (0.2)	<5	N/A	N/A
5 Mormon	18 (0.2)	11 (0.3)	<5	N/A	N/A
*6 Other Christian (text)*	33 (0.3)	37 (0.8)	166 (4.7)	56 (2.6)	15 (0.7)
101/5 Baptist/Evangelical	152 (1.6)	90 (2.1)	42 (1.2)	56 (2.6)	57 (2.8)
102/4 Methodist	121 (1.3)	83 (1.9)	27 (0.8)	58 (2.7)	47 (2.3)
103 Presbyterian	63 (0.7)	24 (0.5)	19 (0.5)	5 (0.2)	<5
104 Pentecostal	42 (0.4)	21 (0.5)	14 (0.4)	<5	<5
105 Quaker	13 (0.1)	10 (0.2)	10 (0.3)	<5	<5
106 Orthodox	26 (0.3)	7 (0.2)	5 (0.1)	<5	<5
107 Lutheran	<5	<5	<5	<5	<5
108 Adventist	8 (0.1)	<5	<5	<5	<5
109 Protestant NOS	40 (0.4)	14 (0.3)	12 (0.3)	5 (0.2)	<5
110 Non-Trinitarian	10 (0.1)	9 (0.2)	<5	<5	<5
111 Non-Conformist	6 (0.1)	5 (0.1)	<5	<5	<5
112 Christian NOS	127 (1.3)	52 (1.2)	46 (1.3)	9 (0.4)	9 (0.4)
7 Judaism	8 (0.1)	5 (0.1)	<5	14 (0.7)	<5
8 Buddhist	32 (0.3)	11 (0.3)	21 (0.6)	17 (0.8)	14 (0.7)
9 Sikh	18 (0.2)	<5	<5	<5	<5
10 Hindu	21 (0.2)	<5	<5	<5	<5
11 Muslim	59 (0.6)	16 (0.4)	10 (0.3)	<5	<5
*Other – (text):*	23 (0.2)	7 (0.2)	6 (0.2)	6 (0.3)	88 (4.3) #
201 Spiritualist	13 (0.1)	<5	<5	7 (0.3)	<5
202 New Age + 205 Pagan *	23 (0.2)	6 (0.1)	6 (0.2)	7 (0.3)	<5
203 Eastern Philosophies	13 (0.1)	<5	<5	<5	<5
204 West Philosophies/ Humanists	21 (0.2)	6 (0.1)	12 (0.3)	8 (0.4)	12 (0.6)
206 NRMs	<5	<5	<5	<5	<5
301 Spiritual (SBNR)	<5	<5	<5	6 (0.3)	<5
302 Belief in Higher Power(s)	49 (0.5)	14 (0.3)	8 (0.2)	<5	5 (0.2)
303 Own/Miscellaneous beliefs	85 (0.9)	16 (0.4)	11 (0.3)	8 (0.4)	16 (0.8)
304 Syncretic	6 (0.1)	<5	<5	<5	<5
401 Agnostic	53 (0.5)	22 (0.5)	17 (0.5)	<5	<5
402 Atheist	72 (0.7)	30 (0.7)	14 (0.4)	<5	<5
501 Niche + 601 Unclassifiable *	27 (0.3)	10 (0.2)	8 (0.2)	<5	10 (0.5)

*Amalgamated due to small cell counts

#Amalgamated Judaism, Sikhism, Hindu, Muslim with Other at this time point due to small cell counts. AN = Data collected during pregnancy; PP = post-partum, i.e. responses at age in years of index child. NRMs = New religious movements; SBNR = spiritual but not religious; NOS = not otherwise stated.

N.B. Agnostics and Atheists are those that have stated such in text form and are additional to Q1 on belief in God or a divine power (Yes, No, Not sure)

**Table 4c.** G1 cohort current beliefs pre- and post-pandemic. Please note that 299 (6.5%) of respondents at 27+ years of age completed the questionnaire after lockdown commenced on 23 ^rd^ March 2020. Cell counts of <5 may include values of zero.

G1 Code/Belief	Index child age *Variable name*	
	**27+ Year** *YPG3040*	**29+ Year** *YPJ6540*
*Total responses*	*N = 4461*	*N=4186*
*Belief in God/Divine Power*		
No	2508 (56.2)	2571 (61.4)
Yes	756 (16.9)	664 (15.9)
Not sure	1197 (26.8)	951 (22.7)
*Faith Type nowadays*	*N= 4424*	*N= 4174*
0 None	2903 (65.6)	3043 (72.9)
1 Church of England	843 (19.0)	578 (13.8)
2 Roman Catholic	161 (3.6)	105 (2.5)
3 Jehovah’s Witness	13 (0.3)	8 (0.2)
5 Baptist/Evangelical	83 (1.9)	94 (2.3)
4 Methodist	47 (1.1)	25 (0.6)
**6 Other Christian text, of** **which:**	**89 (2.0)**	**34 (0.8)**
*[Of which, redistributed to other* *denominations as appropriate]*	***[25]***	***[9]***
103 Presbyterian	<5	<5
104 Pentecostal	7	<5
105 Quaker	6	<5
106 Orthodox	<5	<5
108+109+110 Adventist, Protestant, Non-Trinitarian *	7	5
112 Christian NOS	19	11
7 Judaism	<5	5 (0.1)
8 Buddhist	28 (0.6)	29 (0.7)
9 Sikhism	7 (0.2)	6 (0.1)
10 Hinduism	5 (0.1)	6 (0.1)
11 Muslim	20 (0.5)	21 (0.5)
**14 Other beliefs of which:**	**251 (5.7)**	**223 (5.3)**
*201 Spiritualist*	*59 (23.5)*	*6 (2.7)*
*202 New Age*	*8 (3.2)*	*<5*
*203 + 204 Eastern & West* Philosophies/Humanists *	*10 (4.0)*	*6 (2.7)*
*205 Pagan*	*25 (10.0)*	*28 (12.6)*
*206 NRMs*	*<5*	*<5*
*301 Spiritual*	*43 (17.1)*	*54 (24.2)*
*302 Belief in Higher Power(s)*	*9 (3.6)*	*20 (9.0)*
*303 Own/Miscellaneous beliefs*	*20 (8.0)*	*20 (9.0)*
*401 Agnostic*	*41 (16.3)*	*43 (19.3)*
*402 Atheist*	*<5*	*<5*
501 Niche + 307 Magic *	*<5*	*5 (2.2)*
*601 Unclassifiable*	*6 (2.4)*	*8 (3.6)*

*amalgamated due to small cell counts

Table 5 shows the responses to a question concerning the faith in which they had been brought up (if any). Over a third of G0 mothers and over 40% of their partners were brought up in a different faith to the one they currently had, although the majority of these were brought up in different Christian denominations. Less than 1% were brought up in NMBs, and over 3% in Atheist households. Within the G1 cohort, 42% were brought up in a different faith/denomination.

**Table 5.** Frequency of responses to the question “Were you brought up in this faith (including none)? If not, which faith if any?” Cell counts of <5 may include values of zero.

Were you brought up in this faith (including none)? *Variable name*	Mothers 2020 *Y3060*	Partners 2020 *FPC3060*	G1 27+yrs *YPG3060*
Total responses	N=4689	N= 2133	N=4324
Yes	3175 (67.7)	1264 (59.3)	2506 (58.0)
No, if no which faith if any?	1514 (32.3)	869 (40.7)	1818 (42.0)
0 None	-	-	*343 (7.9)*
1 Church of England	*557 (11.9)*	*368 (17.2)*	*304 (7.0)*
2 Roman Catholic	*138 (2.9)*	*93 (4.4)*	*145 (3.3)*
3 Jehovah’s Witness	*9 (0.2)*	*<5*	*5 (0.1)*
5 Baptist/Evangelical	*24 (0.5)*	*18 (0.8)*	*17 (0.4)*
4 Methodist	*78 (1.7)*	*53 (2.5)*	*27 (0.6)*
103 Presbyterian	*33 (0.7)*	*12 (0.6)*	*<5*
Other denominations amalgamated *	*13 (0.3)*	*6 (3.1)*	*8 (0.2)*
109 Protestant NOS	*7 (0.1)*	*5 (0.2)*	*11 (0.2)*
110 Non-Trinitarian	*8 (0.2)*	*5 (0.2)*	*5 (0.1)*
112 Christian NOS	*163 (3.5)*	*108 (5.1)*	*440 (10.2)*
7 Judaism	*<5*	*<5*	*<5*
8 Buddhism	*<5*	*<5*	*<5*
9 Sikhism	*<5*	*<5*	*<5*
10 Hindu	*<5*	*<5*	*<5*
11 Muslim	*<5*	*<5*	*<5*
All NMBs G0 (201-06; 302-07; 501) * All NMBs G1 (201,204,205,301-04) *	*18 (0.4)*	*6 (0.3)*	*9 (0.2)*
305 Mixed households	*0*	*0*	*19 (0.4)*
306 Personal choice	*0*	*0*	*33 (1.8)*
401 Agnostic	*<5*	*<5*	*5 (0.1)*
402 Atheist	*167 (3.6)*	*70 (3.3)*	*9 (0.2)*
601 Unclassifiable	*<5*	*<5*	*<5*

NMB = non-mainstream beliefs; *amalgamated due to small numbers NOS Not otherwise stated

Over 60% of both mothers and their partners brought their child(ren) up in their current faith (including none). Of those who did not, over 2% of parents were allowing their offspring to make their own choice, but the largest group (over 10% of mothers and slightly more partners) stated they were brought up ‘Atheist’. Over 31% of the G1 cohort were bringing their child up in their current faith (including none) ( Table 6).

**Table 6.** Frequencies in response to the question administered in 2020 when the offspring were aged 27+ years concerning the faith (if any) in which they had/would bring their children up. Cell counts of <5 may include values of zero.

Brought/bringing child(ren) up in current faith (including none)? *Variable name*	Mothers *Y3070*	Partners *FPC3070*	Offspring *YPG3070*
Total responses	N=4701	N=2129	N=4415
Yes, this faith	3177 (67.6)	1335 (62.7)	1401 (31.7)
No, if no what faith if any?:	1524 (32.4)	794 (37.3)	538 (12.2)
Not sure (G1 only)	N/A	N/A	986 (22.3)
N/A (G1 only)	N/A	N/A	1490 (33.7)
0 None (G1 only)	N/A	N/A	*148 (3.3)*
1 Church of England	*124 (2.6)*	*105 (4.9)*	*7 (0.1)*
2 Roman Catholic	*63 (1.3)*	*51 (2.4)*	*10 (0.2)*
3 Jehovah’s Witness	*<5*	*<5*	*<5*
9-11 Sikh, Hindu, Buddhist, Muslim *	*5 (0.1)*	*<5*	*5 (0.1)*
101 Baptist/Evangel	*5 (0.1)*	*5 (0.2)*	*<5*
102 Methodist	*13 (0.3)*	*15 (2.8)*	*<5*
Other denominations amalgamated *	*5 (0.1)*	*8 (0.4)*	*<5*
105 Quaker	*5 (0.1)*	*<5*	*<5*
112 Christian NOS	*66 (1.4)*	*38 (1.8)*	*7 (0.1)*
201 Spiritualist	*<5*	*<5*	*<5*
204 Western Philosophies	*<5*	*<5*	*<5*
301 Spiritual (SBNR)	*<5*	*<5*	*<5*
302 Belief in Higher Power(s)	*<5*	*<5*	*<5*
303 Miscellaneous	*8 (0.2)*	*6 (0.3)*	*<5*
306 Personal choice	*108 (2.3)*	*63 (2.9)*	*93 (2.1)*
401 Agnostic	*<5*	*<5*	*<5*
402 Atheist	*510 (10.8)*	*247 (11.6)*	*<5*
601 Unclassifiable	*<5*	*<5*	*<5*

*amalgamated due to small numbers

When analysing the text data, it would be important to consider the following:

(a) The first question in the RSBB section is ‘Do you believe in God or a Divine Power?’ with tick boxes for Yes/No/Not sure. This has been interpreted as Religious/Atheist/ Agnostic respectively. The text responses have yet to be compared with these answers. For example, it is likely some may have indicated ‘Not sure’ and then provided text indicating some form of belief.
(b) The frequencies to the questions to what extent the respondents considered themselves religious or spiritual are shown in Table 7a for the parent cohorts, pre- and post-pandemic. Unsurprisingly, more mothers than partners considered themselves religious or spiritual, with more of both considering themselves more spiritual than religious. About 31% of mothers and 23% of partners considered religion/spirituality of high or moderate importance to them. Interestingly, those stating ‘very religious’ or ‘not at all religious’ both increased slightly from pre- to post-pandemic, whereas the moderate and slightly religious decreased over the two time points for both sexes. A similar pattern is seen for the extent to which they considered themselves spiritual: but for mothers there was a slight decrease in the very spiritual post-pandemic, and the number of partners who said they were slightly spiritual increased by over 2%. The non-importance of religion/spirituality increased from pre- to post-pandemic timepoints by ~8% in both sexes. It should be noted that it may not necessarily be the same individuals responding at both time points.
(c) Table 7b gives the G1 frequencies for the same questions, prior to (27+ years), and post-pandemic (29+ years). Note that 299 (6.5%) of respondents at 27+ years of age completed the questionnaire after lockdown commenced on 23 ^rd^ March 2020. For both the religious and the spiritual, the numbers stating ‘not at all’ increased post-pandemic. Again, the responses to ‘How important is religion/spirituality to you nowadays?’ resulted in a 7% increase in ‘not at all’ post-pandemic. Again, it may not necessarily be the same individuals responding at both time points.

**Table 7a.** Parent cohort (G0) frequencies to the questions “To what extent do you consider yourself a religious/spiritual person nowadays?” and “How important is religion/spirituality to you nowadays?” responses pre- and post-pandemic.

To what extent do you consider yourself…	Mothers 2020	Mothers 2022	Partners 2020	Partners 2022
**Religious** ( *variable name*)	*Y3210*	*Z5600*	*FPC3210*	*FPD5600*
Total responses	*N=4725*	*N=4642*	*N=2165*	*N=2044*
Very	113 (2.4)	151 (3.3)	46 (2.1)	64 (3.1)
Moderately	653 (13.8)	536 (11.5)	255 (11.8)	175 (8.6)
Slightly	1549 (32.8)	1397 (30.1)	512 (23.6)	447 (21.9)
Not at all	2408 (51.0)	2558 (55.1)	1352 (62.4)	1358 (66.4)
**Spiritual** ( *variable name*)	*Y3220*	*Z5601*	*FPC3220*	*FPD5601*
Total responses	*N=4724*	*N=4641*	*N=2160*	*N=2044*
Very	368 (7.8)	319 (6.9)	99 (4.6)	101 (4.9)
Moderately	937 (19.8)	739 (15.9)	347 (16.1)	213 (10.4)
Slightly	1400 (29.6)	1325 (28.5)	439 (20.3)	452 (22.1)
Not at all	2019 (42.7)	2258 (48.7)	1275 (59.0)	1278 (62.5)
**How important is R/S to you?** ( *variable name*)	*Y3230*	*Z5602*	*FPC3230*	*FPD5602*
Total responses	*N=4725*	*N=4633*	*N=2168*	*N=2046*
Highly	662 (14.0)	549 (11.8)	222 (10.2)	183 (8.9)
Moderately	793 (16.8)	599 (12.9)	284 (13.1)	170 (8.3)
Slightly	1417 (30.0)	1281 (27.6)	476 (22.0)	425 (20.8)
Not important at all	1853 (39.2)	2204 (47.6)	1186 (54.7)	1268 (62.0)

R/S = Religion/Spiritual

**Table 7b.** Offspring cohort (G1) frequencies to the questions “To what extent do you consider yourself a religious/spiritual person nowadays?” and “How important is religion/spirituality to you nowadays?” responses pre- and post-pandemic. Please note that 299 (6.5%) of respondents at 27+ years of age completed the questionnaire after lockdown commenced on 23 ^rd^ March 2020.

To what extent do you consider yourself….	YPG 27+ years	YPJ 29+ years
**Religious** ( *variable name*)	*YPG3210*	*YPJ6590*
*Total*	*N=4432*	*N=4166*
Very	56 (1.3)	57 (1.4)
Moderately	198 (4.5)	150 (3.6)
Slightly	732 (16.5)	555 (13.3)
Not at all	3446 (77.8)	3404 (81.7)
**Spiritual** ( *variable name*)	*YPG3220*	*YPJ6591*
*Total*	*N=4428*	*N=4172*
Very	185 (4.2)	146 (3.5)
Moderately	494 (11.2)	364 (8.7)
Slightly	1177 (26.6)	1010 (24.2)
Not at all	2572 (58.1)	2652 (63.6)
**How important is R/S to you?** ( *variable name*)	*YPG3230*	*YPJ6592*
*Total*	*N=4428*	*N=4161*
Highly	250 (5.6)	201 (4.8)
Moderately	395 (8.9)	285 (6.8)
Slightly	968 (21.9)	750 (18.0)
Not important at all	2815 (63.6)	2925 (70.3)

R/S = Religion/Spirituality

### Discussion points

We found an eclectic mix of Christian denominations, world religions, NMBs and non-religious world views within ALSPAC. These frequencies are comparable with regional and national statistics.

In this paper we have concentrated on explaining the system used to code text data describing these beliefs. Future analyses could include duration of belief; intergenerational differences; whether NMBs fall in and out of fashion (e.g., Jedi Knight with over 2300 reported in Bristol in the 2011 census but none in the 2021 census - the Jedi anomaly was due to a campaign in 2011 to encourage people to state this belief in the Census – see Table 2).

As Lois Lee (2018) put it: “Nonreligious worldviews are slippery fish – and nonreligious people often struggle as much as anyone else to put them into words.” This is evident from the diverse and sometimes vague responses coded under 303 (Miscellaneous and ‘own’ beliefs).

As alluded to above, the spiritual but not religious (SBNR) and ‘spiritualist’ groups pose an interesting challenge in themselves. The meaning of ‘spiritual’ has changed, especially in the last few decades, from the deeply religious to those seeking inner harmony, peace or meaning and purpose in life through means other than organised religion. More and more people label themselves thus, both in ALSPAC and, for example, as reported in the USA ( Lipka and Gecewitz, 2017) where those professing to be SBNR rose from 19% in 2012 to 27% in 2017; whilst the religious and spiritual decreased from 59% to 48% over the two time points. 

Koenig *et al.* (2023) have given helpful definitions for the ‘religious’ (with sub-categories); the ‘spiritual’; and the ‘secular humanists’ for the purposes of research. This latter group which the authors describe as “a way of being in the world that provides significance, meaning and purpose, but without transcendence” and includes atheism, agnosticism, naturalism and materialism. Consideration of these construct definitions which are “largely agreed-upon, distinct, measurable and do not overlap with health states…” ( Koenig *et al.*, 2023) may prove helpful in future research.

In conclusion, those not affiliated with formal religions including atheists, agnostics and those with NMBs/non-religious beliefs have complex and far from clear cut views. There is scope to examine these religious ‘nones’ as they incorporate many diverse beliefs including the existence of supernatural forces, existential and metaphysical beliefs about life and death, the meaning of life and worldviews. These beliefs exist across a wide range of sociodemographic contexts, personal and social identities ( Lee, 2014). As Lee and colleagues suggest, rather than labelling people as believers and unbelievers, … “we can start to imagine and document more ‘hybrid’ configurations in which a single individual might have, say, a materialist conception of life and the afterlife, derive other existential beliefs about what matters in life from a Catholic background, and hold ethical principles informed by a degree of relativistic agnosticism and the sense that humans should not exert undue influence over the lives of others” ( Lee *et al.*, 2017).

***Strengths/ limitations***

One of the main strengths of the ALSPAC study is that almost identical religious/spiritual beliefs and behaviours (RSBB) data have been collected on five occasions over a 30-year period in the mother and partner cohorts, from prenatally (thus avoiding any effects of a life event (birth of the child) in parental RSBB); to just after the COVID-19 pandemic. Identical data have been collected on the offspring cohort since age 27. Data collection was prospective and unlikely to result in recall bias. However, the questions or scales used in the study have a bias towards the religious and/or spiritual rather than teasing out the beliefs of the nonreligious.

The original county of Avon comprised the city of Bristol along with the counties of North Somerset, South Gloucestershire and parts of Bath & Northeast Somerset (BANES) with both urban and rural areas, and small towns such as Weston-Super-Mare, Clevedon, Portishead and Yate ( Ellis *et al.*, 2022). Few non-white participants and/or adherents to the world religions lived in rural districts outside Bristol in 1991 and since the question on religious faith was not included in the Census until 2001, it is difficult to compare ALSPAC directly with Avon or national data at that time. For Census data reported here, Avon had been disbanded and split between the counties mentioned above, and the ‘Avon’ figures exclude those areas in BANES that were previously within Avon.

As with all longitudinal studies, attrition is a problem. In ALSPAC this has been higher for those who experienced more adversity at the time of the index pregnancy. Subsequently, attrition is due mainly to mortality, change of address (and consequent loss to follow up), as well as a reluctance to stay involved (although formal withdrawal from the study is quite rare) and shown to be non-random ( Boyd *et al.*, 2013; Cornish *et al.*, 2021; Fraser *et al.*, 2013; Fernández-Sanlés *et al.*, 2022; Taylor *et al.*, 2018). Therefore, selection bias becomes a problem such that the current participants are those less socio-economically deprived, have a higher educational attainment and were initially more likely to profess a faith compared with those no longer taking part. However, analyses of the ways in which such attrition may bias results is likely to be minimal ( Morgan *et al.*, 2022).

We are unaware of any other longitudinal cohort that has collected similar data over such a long period of time in two generations. Consequently, any results that are published with the ALSPAC data are unlikely, at this stage, to be able to be tested for replication.

## Data Availability

ALSPAC data access is through a system of managed open access. The steps below highlight how to apply for access to the data included in this paper and all other ALSPAC data. Note that variable names are included in the tables within this paper. Please read the ALSPAC access policy ( http://www.bristol.ac.uk/media-library/sites/alspac/documents/researchers/data-access/ALSPAC_Access_Policy.pdf) which describes the process of accessing the data and biological samples in detail, and outlines the costs associated with doing so. 1. You may also find it useful to browse our fully searchable research proposals database ( https://proposals.epi.bristol.ac.uk/), which lists all research projects that have been approved since April 2011. 2. Please submit your research proposal ( https://proposals.epi.bristol.ac.uk/) for consideration by the ALSPAC Executive Committee using the online process. You will receive a response within 10 working days to advise you whether your proposal has been approved. If you have any questions about accessing data, please email: alspac-data@bristol.ac.uk (data) or bbl-info@bristol.ac.uk (samples). The ALSPAC data management plan ( http://www.bristol.ac.uk/media-library/sites/alspac/documents/researchers/data-access/alspac-data-management-plan.pdf) describes in detail the policy regarding data sharing, which is through a system of managed open access. OSF: Appendix 1: Coding schema, https://doi.org/10.17605/OSF.IO/ZVB2A ( Iles-Caven, 2023a) This project contains the following extended data: App1Religion Recoding 2023PublicV9.docx OSF: Appendix 2: Glossary of Beliefs, https://doi.org/10.17605/OSF.IO/DK9RC ( Iles-Caven, 2023b) This project contains the following extended data: App2Glossary of beliefs.docx Data are available under the terms of the Creative Commons Attribution 4.0 International Public License ( CC-By Attribution 4.0 International)
