# Supplementary material for: Coding definitions of participant religious, non-religious and spiritual beliefs in the Avon Longitudinal Study of Parents & Children (ALSPAC)
Source: Wellcome Open Res. 2023 Nov 16;8:528. [Version 1] doi: 10.12688/wellcomeopenres.20209.1 (PMC11234077; doi:10.12688/wellcomeopenres.20209.1)
Supplement: Peer review history

# Reviewer report: Reviewer response for version 1

**DOI:** 10.21956/wellcomeopenres.22376.r87838

**Published:** 2024-07-09

**Authors:** Roberto Cipriani

**Affiliations:** 1 Roma Tre University, Rome, Lazio, Italy

The paper is intriguing and delves deeply into the ongoing debate about religious beliefs and the controversy between religious and spiritual in religious phenomenology. The presentation is highly detailed, although it might be overly so. This level of detail sometimes causes confusion, making it difficult for the reader to follow all the sociological discussions. It's especially challenging to analyze and compare different investigations, typologies, denominations, and definitions.

Are sufficient details of methods and materials provided to allow replication by others?

Yes

Is the rationale for creating the dataset(s) clearly described?

Yes

Are the datasets clearly presented in a useable and accessible format?

Yes

Are the protocols appropriate and is the work technically sound?

Yes

Reviewer Expertise:

Sociology of religion

I confirm that I have read this submission and believe that I have an appropriate level of expertise to confirm that it is of an acceptable scientific standard.

# Reviewer report: Reviewer response for version 1

**DOI:** 10.21956/wellcomeopenres.22376.r75081

**Published:** 2024-03-14

**Authors:** Rodrigo Toniol

**Affiliations:** 1 University of Rio de Janeiro, Rio de Janeiro, Brazil

The article "Coding definitions of participant religious, non-religious and spiritual beliefs in the Avon Longitudinal Study of Parents & Children (ALSPAC)" presents a set of original and pertinent reflections both for handling the volume of ALSPAC data and for a more specific debate involving demographic health data. The article aims to establish groupings between the categories mobilized by the survey participants to express their religious practices. The proposal is challenging insofar as ALSPAC consists of a longitudinal survey that captures variations in the religious practices of the people surveyed and how religions are socially recognized. In Latin America, for example, the case of the Census surveys in Brazil is widely known, which between the 1990s and 2000s found a variation in the forms of self-identification of Pentecostal believers: if in the 1990 census, they presented themselves as believers of a specific religious denomination, such as the Universal Church of the Kingdom of God, in the 2000s they began to identify themselves simply as "evangelicals." Behind this variation was a social phenomenon only later identified by experts in the field: the choice of the broader term "evangelicals" was related to more general transformations in the Brazilian religious field, in which believers began to move more between different religious denominations. 

 The article in question, therefore, addresses the issue of ways of producing frames to describe a wide variety of religious actors. The methodology is explicit, and the results are well outlined. The authors, however, refrain from dialoguing with an immense accumulation of debates on the subject of categories of definition and ways of classifying religion by social scientists. To open a dialogue with this literature would overextend the article's scope and go beyond its aims. Nevertheless, this theoretical tradition is worth mentioning. 

 Finally, the grouping proposed by the authors may be helpful for other researchers who also look at the data from the Avon Longitudinal Study of Parents & Children.

Are sufficient details of methods and materials provided to allow replication by others?

Yes

Is the rationale for creating the dataset(s) clearly described?

Yes

Are the datasets clearly presented in a useable and accessible format?

Yes

Are the protocols appropriate and is the work technically sound?

Yes

Reviewer Expertise:

Anthropology; Religion; Spirituality; Health; Qualitative Research

I confirm that I have read this submission and believe that I have an appropriate level of expertise to confirm that it is of an acceptable scientific standard.
